# Improvement of Tuberculosis Laboratory Capacity on Pemba Island, Zanzibar: A Health Cooperation Project

**DOI:** 10.1371/journal.pone.0044109

**Published:** 2012-08-27

**Authors:** Maria G. Paglia, Nazario Bevilacqua, Haji Said Haji, Francesco Vairo, Enrico Girardi, Emanuele Nicastri, Juma Muhsin, Vincenzo Racalbuto, Mohammed S. Jiddawi, Giuseppe Ippolito

**Affiliations:** 1 Microbiology Laboratory, Epidemiology and Clinical Departments, National Institute for Infectious Diseases “Lazzaro Spallanzani”, Rome, Italy; 2 Public Health Laboratory–Ivo De Carneri, Chake Chake, Pemba Island, Zanzibar, Tanzania; 3 National Tuberculosis and Leprosy Programme, Ministry of Health and Social Welfare, Unguja Island, Zanzibar, Tanzania; 4 Italian Cooperation Agency, Italian Ministry of Foreign Affairs, Rome, Italy; 5 Ministry of Health and Social Welfare, Unguja Island, Zanzibar, Tanzania; Johns Hopkins University School of Medicine, United States of America

## Abstract

Low-income countries with high Tuberculosis burden have few reference laboratories able to perform TB culture. In 2006, the Zanzibar National TB Control Programme planned to decentralize TB diagnostics. The Italian Cooperation Agency with the scientific support of the “L. Spallanzani” National Institute for Infectious Diseases sustained the project through the implementation of a TB reference laboratory in a low-income country with a high prevalence of TB. The implementation steps were: 1) TB laboratory design according to the WHO standards; 2) laboratory equipment and reagent supplies for microscopy, cultures, and identification; 3) on-the-job training of the local staff; 4) web- and telemedicine-based supervision.

From April 2007 to December 2010, 921 sputum samples were received from 40 peripheral laboratories: 120 TB cases were diagnosed. Of all the smear-positive cases, 74.2% were culture-positive. During the year 2010, the smear positive to culture positive rate increased up to 100%.

In March 20, 2010 the Ministry of Health and Social Welfare of Zanzibar officially recognized the Public Health Laboratory- Ivo de Carneri as the National TB Reference Laboratory for the Zanzibar Archipelago.

An advanced TB laboratory can represent a low cost solution to strengthen the TB diagnosis, to provide capacity building and mid-term sustainability.

## Introduction

Worldwide, 13.7 million people live with tuberculosis with an incidence of 9.4 million new cases per year [1]. The disease kills more young people and adults than any other infectious disease. Tanzania is one of the 22 high-burden countries with respect to the number of incident tuberculosis (TB) cases [1].

Currently, TB culture laboratories in resource-poor countries often lack adequate infrastructures and have inadequate or outdated equipment, and poor biosafety measures with a scarcity of human and financial resources. Unfortunately, most of the resource-poor regions of the world with high burdens of TB have very few reference laboratories capable of reliably performing TB culture and drugs sensitivity tests.

Records from the National Tuberculosis and Leprosy Program of Tanzania show that since 1983, the number of notified TB cases has increased almost six-fold, from 11,753 to 65,665 in 2004 [2]. In Tanzania the current TB incidence is 153 per 100,000 population: 59 per 100,000 are new smear positive cases [1], [3].

Delays in both diagnosis and treatment is a major impeding factor in the control of tuberculosis [4], [5]. In Mwanza (Tanzania), a study found that about 80% of patients reported to health facilities after more than thirty days from the onset of symptoms, with a mean delay of 162 days [6].

In the Zanzibar archipelago, that comprises Unguja and pemba islands on 2,332 square kilometres, the tuberculosis prevalence is estimated around 30×100,000 population [7], [8], the general prevalence of HIV/AIDS in the sexually active population is 0.6% [8].

Pemba Island is a small Island located 50 km from the north-west coast of Unguja, the main Island of the Zanzibar archipelago. The Population of Pemba is 361.000 inhabitants from the 2002 Census.

**Figure 1:**
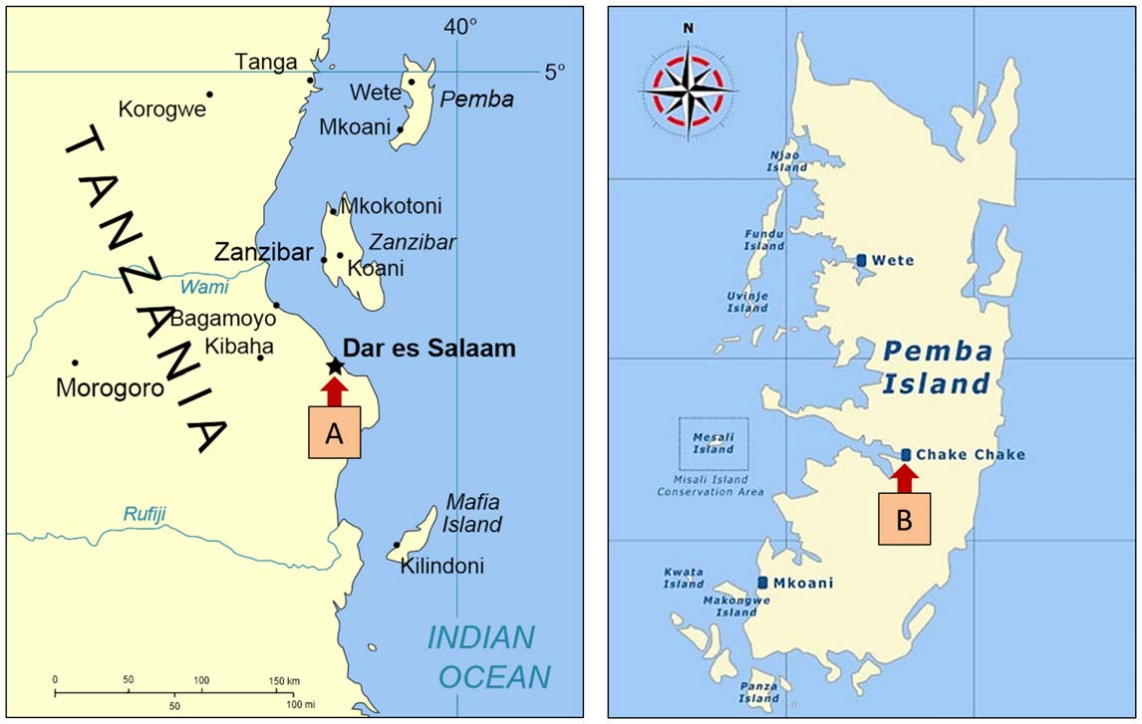
Map illustrating the sites of the TB laboratories. A: Central TB Reference Laboratory; B: Pemba Reference Laboratory.

In 2005, within the Zanzibar National Tuberculosis and Leprosy Program, TB diagnostic capacity in Zanzibar was limited to smear microscopy, performed in 40 peripheral laboratories (26 on Unguja Island and 14 on Pemba Island) without adequate quality assurance (QA) programs. All clinical specimens requiring culture and drug susceptibility testing (DST) were sent to the Central Tuberculosis Reference Laboratory (CTRL) of the Muhimbili Hospital in Dar es Salaam, around 50 km from Unguja Island (Figure 1).

The laboratory where the interventions took place is part of the Public Health Laboratory-Ivo de Carneri (PHL-IdC). The facility is located on Pemba Island and it represents a successful public-private partnership between the Italian non-governmental organization, the Ivo de Carneri Foundation, and the Ministry of Health and Social Welfare (MoHSW) of Zanzibar, which has PHL-IdC in its organigram.

Currently, the laboratory covers the all Zanzibar Archipelago with a population of 981,754 inhabitants (Census 2002).

This article describes the start-up and implementation of a reference laboratory for the diagnosis of tuberculosis at the premises of the PHL-IdC on Pemba island.

## Results

In April 2007, a TB section on the premises of the PHL-IdC on Pemba Island of the Zanzibar Archipelago was initiated to perform sputum smear microscopy, TB solid culture and identification of *Mycobacterium tuberculosis* by biochemical assays. The human and economical contributions by the Italian Cooperation Agency and the technical support of the “Lazzaro Spallanzani” National Institute of Infectious Diseases jointly with the Zanzibar MoHSW, supported the design, building and implementation of the TB research and care laboratory during a non-consecutive period of 9 months stratified into four different phases detailed in the Methodology section. Both the TB isolation rate and the contamination rates of solid culture LJ media were available during the fourth phase, in April 2007.

Over a period of forty-six months, from April 2007 to December 2010, a total of 921 sputum samples were sent to the TB section of the PHL-IdC by 14 peripheral laboratories of Pemba Island, and since July 2009, by 26 peripheral laboratories of Unguja Island. The specimens were processed for culture using Lowenstein -Jensen media only. All samples were eligible for testing, with a mean of 20,4 (+/− 16.1%) processed samples per month.

One hundred and twenty-one pulmonary TB cases were diagnosed. Considering 115 smear-positive cases from April 2007 to December 2010, 84 samples (73.0%) were culture-positive. During the 2010 year, 18 smear-positive cases resulted all as culture-positive and the smear-positive to culture-positive rate increased up to 100%. The smear-positive to culture-negative rate, calculated as smear-positive/culture-negative divided by the total number of cultures, was 3.4% (31/920) and 0% in the years 2007–2010 and in 2010, respectively. The rate of smear-negative to culture-positive results, calculated as smear-negative/culture-positive divided by the total culture-positives, was 6.7% (6/90) and 10% (2/20) in the years 2007–2010 and in 2010, respectively.

The investment required to upgrade TB diagnostic services was of 36,960 USD, including 6,600 USD for TB diagnostic instruments (combined fridge, orbital shaker and others), 15,840 USD for reagents and consumables for two years, 1,320 USD for training the local staff and € 13,200 USD for expert missions expenditures.

In March 20, 2010 the MoHSW of Zanzibar officially recognized the PHL-IdC as the National TB Reference Laboratory for the Zanzibar Archipelago.

## Discussion

Laboratory services are one of the most neglected areas of health care provision in sub-Saharan Africa and are disproportionately affected by staff shortage, poor communication, inadequate equipment, low morale, and lack of training that impinge on all those involved in delivering health care in poorer African countries.

In our experience, the set-up of an intermediate level TB laboratory has been achieved with low costs, within a relatively short period of time in a low-resource setting. Since 2010, the TB laboratory at PHL-IdC has been budgeted under the Zanzibar Tuberculosis and Leprosy Programme (NTLP).

These results depended on the active cooperation between the local health authorities, the private component of the PHL-IDC, the Italian Cooperation Agency and the Spallanzani's technical support. Transferring high quality technology to resource-limited settings is an achievable result: the scaling-up of a TB laboratory in Perù from 1996 to 2005 [9], and the implementation of a national reference laboratory with appropriate biosafety containment in Lesotho from 2006 to 2008 [10], are recent models of integration between cooperation agencies and national health authorities. The concept of strengthening laboratories that integrate multiple major diseases at each level of a health service in malaria and HIV infection has been a clear priority in Malaria and HIV National Control Programs in Tanzania and Nigeria but only more recently the TB laboratory surging capacity has become a real challenge for the African countries [11], [12].

Although the initial start-up of the TB laboratory in Pemba was complete, Zanzibar geographical, economic, and climatic constraints require further implementations to avoid supply and reagent shortage, inappropriate transportation of biological samples and to provide an optimal referral system and long- term sustainability.

Differently from our results, in Lesotho, Paramasivan, CN et al found a smear-negative to culture-positive rate and a smear-positive to culture-negative rate of 49.9% and 1.6%, respectively [10]. The lower prevalence rate of smear-negative to culture-positive samples on Pemba (6.3%) than in Lesotho from 2007 to 2010 could be explained by different factors. First, the 23.6% HIV prevalence rate in Lesotho adults is much higher than the 0.6% prevalence reported in Zanzibar and could contribute to the reduction of the TB smear-positive rate commonly found in HIV-TB co-infected patients [8], [13]. Second, the preliminary results found in Zanzibar on a limited number of processed samples need to be confirmed on a larger population of patients and in a long-term prospective trial. However, in Pemba during the year 2010, the smear-negative to culture-positive rate and the smear-positive to culture-negative rate continued the favourable trend, improving up to 10% and 0%, respectively. The high rate of smear positive to culture negative before 2010 could be attributed to low personnel performance. As the staff experience improved over the years, all positive direct tests were confirmed by growth of mycobacterial culture.

The project conducted on Pemba, Zanzibar, represents an integrated model of health cooperation in a low-resource setting implementing a high-quality TB diagnostic capacity according to the current WHO recommendations on TB [14]. The need of integrating the TB laboratory of PHL-IdC into the national strategic plan for the development of comprehensive TB laboratory systems has been a priority over the years. Further efforts within the Zanzibar MoHSW, the Cooperation Partner Agencies, the WHO and the Stop TB program, the Global Laboratory Initiative, and the New Diagnostics Working Group are needed to provide long-term sustainability of the laboratory capacity for the TB diagnostic services [15]. The public-private mix (PPM) seems to be one of the correct answers to face the 30% funding gap of TB control by the NTLP of Tanzania [3] and to deliver high quality TB services in line with the international standards.

In the literature there are very few reports providing the costs of scaling up of a TB laboratory in low-resource settings. To implement a TB laboratory in the capital of the Lesotho, Paramasivan CN et al. reported that the total investment costs were less than 550,000 USD [10]. Hepple P et al. did not mention the economic burden of the TB culture performed on sputum samples from patients attending four Medicins Sans Frontieres clinics in southern Sudan [16]. All these experiences are different in terms of objectives, target population, locations and methods, however the interventions shared similar constraints and challenges to achieve the common goal of the surging capacity building on TB diagnosis in Africa. According with this perspective, the overall investment costs to realize the TB laboratory at PHL-IdC should be considered affordable. The amount of around 37,000 USD is reasonably low in comparison with the obtained results: the implementation of a TB laboratory in a remote area, the mid-term sustainability since 2007, and the recognition as the National TB Reference Laboratory for the Zanzibar Archipelago, in 2010.

The project, carried out through the financial support of the Italian Cooperation Agency, has demonstrated that the establishment of an advanced TB laboratory can represent a low-cost solution to strengthen TB diagnosis in Zanzibar, to provide capacity building of the staff and give mid-term sustainability to the process.

## Methods

### Laboratory organization

All the phases regarding the implementation of the laboratory were discussed and agreed upon with the local health authorities, the MoHSW and the NTLP of Zanzibar. The cooperation activities were conducted from April 2006 to October 2008.

**Figure 2:**
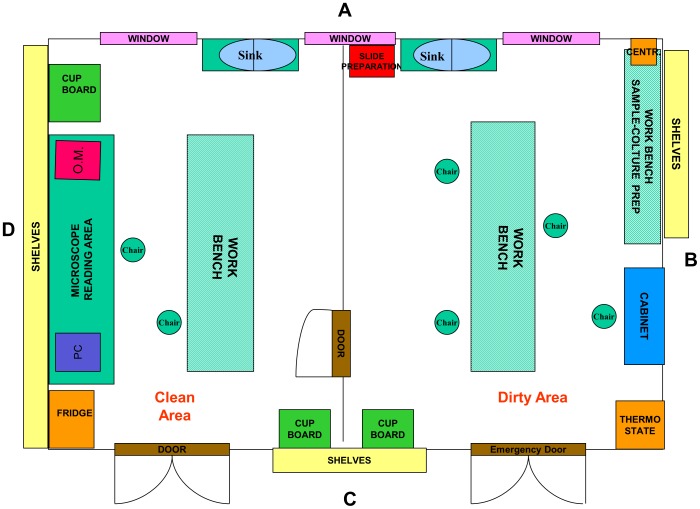
Layout of TB laboratory.

**In the first phase**, which covered the period from June to July 2006, the main steps were: i) identification of suitable space to be converted into the TB laboratory; ii) cleaning and checking the existing useful materials; iii) designing the layout according to WHO standards (Figure 2); testing and validation of a biosafety level II cabinet together with one centrifuge, one incubator, and one microcentrifuge. **In the second phase**, between August and December 2006, the laboratory was equipped with a light microscope, one incubator, one combined fridge and one orbital shaker. All the reagents and disposables for sputum microscopy were supplied. **In the third phase**, from November 2006 to January 2007, laboratory personnel was trained in smear microscopy and solid culture on Lowenstein-Jensen (LJ) media. In particular, one staff member was identified and trained for a period of three months in direct microscopy, culture and drug susceptibility testing in the CTRL. **In the fourth phase**, from March to November 2007, plus one additional visit on October 2008, human resource capacity building and reinforcement were performed by teaching, training, monitoring and mentoring by internet.

### Diagnostic methods

Sputum samples were analyzed from a population of non-hospitalized patients.

Direct smears were prepared and stained using the hot Ziehl-Neelsen (ZN) method. The remaining quota of the concentrated specimens was then decontaminated with 4% NaOH, and inoculated on two Lowenstein-Jensen slants (one tube containing glycerol, and a second tube with pyruvate) and incubated at 37°C for eight weeks, or until growth was detected. The presence of mycobacteria was confirmed by ZN smear for positive cultures, and strains were further identified using the Niacin test.

Internal Quality Assurance system was established for microscopy procedures. Positive and negative smears were prepared for internal quality control. Positive control smears were prepared from a known AFB smear positive patient with 1+ score. Negative control smears were prepared from a culture plate of E. coli growing on a MacConkey agar plate. A visibly turbid suspension in water was prepared. A drop of suspension was placed in the center of each slide prepared. Slides were allowed to air dry and labeled as “Negative”. To evaluate the quality of ZN staining obtained daily, the positive and negative control slides were read before the daily samples.

For External Quality Assurance, there was no system in place at the time of the intervention. The system has been implemented by the TB National Programme in 2011 for microscopy and cultures.

The program carried out on the premises of PHL-IdC could not be classified as “human subjects research”. Since it just implemented and upgraded the TB diagnosis as standard of care involving the secondary use of existing data and thus was exempt from IRB review.

Statistical analysis was carried out by using descriptive statistics.
